# EPOCA Tele-Monitoring System for Older Adults at High Risk of Hospitalization: Budget Impact and Cost-Effectiveness Analysis

**DOI:** 10.2196/80302

**Published:** 2026-05-14

**Authors:** Henri Leleu, Damien Testa, Mireille Dutech, Etienne Minvielle, Elise Cabanes

**Affiliations:** 1 Public Health Expertise, 10 boulevard de Sébastopol, Paris, 75004, France, 33 1 85 09 09 49; 2 Epoca U&I, Nanterre, France; 3 Institut Polytechnique de Paris, Centre de Recherche en Gestion de l’Ecole Polytechnique (I3-CRG, UMR CNRS 9217), Palaiseau, France; 4 Département Interdisciplinaire d’Organisation du Parcours Patient (DIOPP), Gustave Roussy Cancer Centre, Villejuif, France

**Keywords:** budget impact analysis, cost-effectiveness, older adults, polypathology, patient remote monitoring

## Abstract

**Background:**

France’s aging population faces high rates of chronic illness, multimorbidity, and avoidable hospitalizations, placing pressure on an already strained health care system. Remote monitoring systems have shown promise in improving care coordination and reducing acute care use.

**Objective:**

The objective of this study was to assess the cost-effectiveness of the EPOCA remote monitoring system, implemented within the Vigie-Age framework, compared to the standard of care for older adults with multiple chronic conditions.

**Methods:**

Using data from the Vigie-Age Article 51 pilot study (with 722 participants, including 408 participants with long-term follow-up), a cost-utility analysis was conducted over a 10-year lifetime horizon. A Markov model with daily cycles simulated transitions across health states: at home, emergency department visits, hospitalization, and death. Analyses were conducted from both the French National Health Insurance (NHI) and collective perspectives. Direct medical costs, including hospital, outpatient, and intervention costs, were included. Health outcomes were measured in quality-adjusted life years (QALYs). Deterministic and probabilistic sensitivity analyses assessed model robustness.

**Results:**

EPOCA was associated with a reduction in emergency department visits by 54% and in hospitalizations by 46%, cutting the average hospital stay from 55.6 (SD 51.7) to 30.6 (SD 27.8) days. Total costs per patient were €29,165 (EUR €1=US $1.13) with EPOCA and €39,929 for standard of care, representing a €10,764 saving from the collective perspective and a €7,430 saving from the NHI perspective. EPOCA yielded 0.04 additional QALYs and remained cost-saving even at higher program costs. Sensitivity analyses confirmed the robustness of the results. EPOCA had a 90% probability of being dominant and a 95% probability of being cost-effective at a €30,000 per QALY threshold.

**Conclusions:**

On the basis of currently available evidence, EPOCA may be a cost-effective strategy for older patients at high risk of hospitalization. It could reduce health care use while improving outcomes, supporting its integration into national older adult care pathways and reimbursement by the French NHI.

## Introduction

France is undergoing a profound demographic transition marked by a growing older adult population. As of 2024, 10% of the population, equivalent to 7 million individuals, is aged ≥75 years, and this number is expected to rise to 16% by 2050, representing 12 million individuals [1,2].

Chronic diseases are highly prevalent among older adults in France, with 91% of individuals aged ≥75 years presenting at least 1 chronic condition or ongoing treatment. Multimorbidity is also a defining feature of aging: 47% of men and 36% of women aged ≥75 years live with 2 or more chronic diseases, and up to 26% of men and 17% of women in this age group have 3 or more chronic diseases [3]. Aging also significantly increases the risk of loss of independence. Although most older adults remain autonomous until late in life, the share of older adults receiving the French “Allocation Personnalisée d’Autonomie,” a marker of dependency, rises from 2% among adults aged 70 to 74 years to approximately 50% among those aged 90 to 94 years and to three-quarters among those aged ≥95 years [4]. Finally, increasing age is accompanied by increased risks of social isolation, particularly among women. More than half of women aged 85 years live alone, compared to just 25% of men [4].

These demographic shifts place immense pressure on France’s health care infrastructure, which is already strained by shortages of geriatricians and a declining number of general practitioners (GPs). The result is a fragmented and reactive health care system, where emergency departments (EDs) and inpatient services often serve as the default response to complex geriatric needs. Approximately 40% of those aged ≥80 years in France are hospitalized annually, with an average duration of approximately 25 days per patient. It is estimated that more than 30% of these hospitalizations could have been avoided, with similar figures for ED visits [5,6]. These trends underline the urgent need for scalable, home-based care solutions tailored to the evolving needs of an aging, increasingly dependent population.

Remote monitoring systems (RMSs) have gained international traction as a promising solution for managing older patients with chronic and complex health needs. Existing RMSs vary widely in structure and technology but generally include combinations of wearable sensors, mobile health apps, and care coordination frameworks [7-14]. These RMSs are typically multidisciplinary, involving nurses, hospital physicians, geriatricians, and GPs, often with social support services integrated. Most of these RMSs have demonstrated reductions in hospitalizations and emergency visits [9-11,13,14].

Launched in 2019, the Vigie-Age framework is a multidisciplinary hospital-community initiative designed to provide comprehensive, coordinated care for older adults with multiple chronic conditions. Its primary objectives are to enhance quality of life and reduce avoidable hospitalizations through better continuity of care. This framework includes EPOCA, an RMS that is a human-centered connected telemedicine solution offering secure and continuous home support for complex older patients (with multiple comorbidities, loss of autonomy, and/or dependency). It is built around a 24×7 tele-monitoring platform that enables real-time clinical oversight, rapid response to health deterioration, and seamless coordination among health care professionals. The system integrates two core technological components: (1) connected devices, such as a wristband, for transmitting health data or emergency alerts, and (2) a centralized RMS coordination hub staffed by trained nurses and physicians, who engage directly with patients and caregivers when needed. Hospital geriatricians or GPs play a central role in the framework, offering ongoing medical supervision via tele-consultations and tele-expertise and maintaining active communication with community health care teams. In parallel, community-based nurses conduct regular home visits, monitor patient conditions, and deliver essential care services.

Vigie-Age has been included in the French “Article 51” experimental framework launched in 2018 to promote innovation in health care delivery and financing. Article 51 enables pilot studies of interventions that aim to improve care coordination, efficiency, and patient outcomes through new organizational models and payment structures. These pilots are evaluated for their clinical impact, economic sustainability, and potential for national scale-up, providing valuable data.

This study aimed to assess the cost-effectiveness of the Vigie-Age framework, including the EPOCA RMS, compared to standard of care (SOC), focusing on reductions in hospitalizations, emergency visits, and overall health care costs and using real-world data from the Article 51 evaluation of the Vigie-Age framework.

## Methods

### Data Source

This cost-effectiveness analysis is primarily based on data from the Vigie-Age Article 51 pilot study. Conducted prospectively between 2022 and 2024 across 4 hospitals in France, the study enrolled 722 participants. Eligibility criteria included individuals aged ≥70 years with multiple chronic conditions, a history of recent decompensations, and a high risk of further clinical deterioration.

The study population was composed of 36% (263/722) men, with a mean age of 87.9 (SD 7.1) years. Participants experienced an average of 2.0 (SD 1.4) overnight hospitalizations in the 6 months preceding study inclusion. Approximately 70% (499/722) of participants were recruited either during a hospitalization or from a community-based setting, while the remaining 30% (223/722) were included following an ED visit.

The pilot study assessed outcomes related to clinical efficacy (hospitalizations and ED visits), health care resource use, and associated costs. These outcomes were derived from French National Health Insurance (NHI) reimbursement data and compared between the study follow-up period and a similar period preceding inclusion. Among the 408 participants who received the EPOCA RMS intervention, outcome data were available for 269 (66%) individuals with sufficient follow-up, with a mean observation period of 7.5 months.

### Perspective, Population, Intervention, and Comparator

This cost-effectiveness evaluation was conducted from 2 complementary perspectives, both including only direct medical costs. The first was a payer perspective, focusing on health care expenditures paid by the NHI. The second was a collective perspective, in line with the recommendations of the Haute Autorité de Santé [15], which includes all costs regardless of the payer.

The analysis was conducted on a population corresponding to participants enrolled in the Vigie-Age Article 51 study. The EPOCA RMS intervention was compared to SOC, defined as the same participants’ preinclusion health care use and costs, measured over a comparable baseline window prior to program entry. The SOC for this high-risk, older, polymorbid population was defined as usual care provided by the French national health system, consisting of routine community and primary care services (GP follow-up, home-based care, and social support). Crucially, this usual care lacked the structured, proactive tele-monitoring and coordinated case management features that characterize the EPOCA intervention.

### Model Structure

Due to the limited follow-up duration in the Vigie-Age Article 51 pilot study, a de novo Markov model was developed in Excel (version 16.108.3; Microsoft; available on demand) to extrapolate the long-term clinical and economic outcomes of the EPOCA intervention over a lifetime horizon, defined as 10 years based on the average age of the target population. The model used daily cycles and incorporated the following health states: stable condition at home, ED visits, hospitalization (either scheduled or via an emergency visit), and death (all-cause mortality).

The selected health states reflect key events from the Article 51 study: ED visits and hospitalization. Patients start in either the ED or at home; those at home may be hospitalized or visit the ED. In the ED, patients can return home or be hospitalized, while hospitalized patients can only return home. Death is possible at any stage. Hospitalization is associated with disutility, and costs are incurred at every stage, including outpatient care for patients at home.

Transition probabilities for hospitalizations and ED visits were derived from the Vigie-Age Article 51 pilot study. Model parameter estimations incorporated data from all patients included in the Vigie-Age Article 51 pilot study who had a minimum of 1 month of follow-up.

The model also accounted for the recruitment setting, with patients enrolled in the EPOCA intervention during an ED visit avoiding immediate hospitalization, whereas similar patients under SOC could proceed to inpatient admission. Model parameters are detailed in Table 1.

**Table 1.** Model parameters.

Parameters	Values, mean (SD)	95% CI in PSA^a^/DSA^b^	Distribution	Source
Transition probabilities				
SOC^c^				
ED^d^ entry (%)	22	Not included	Not applicable	Art 51^e^
Monthly probability of hospitalization	1.9 (0.04)	1.8-2.0	Normal	Art 51
Monthly probability of ED visits	3.5 (0.05)	3.4-3.6	Normal	Art 51
Proportion of hospitalizations following ED visits (%)	71	64-79	Beta	Art 51
Length of stay (days)	7.7 (6.4)	6.2-9.2	Normal	Art 51
EPOCA				
ED entry (%)	0	Not included	Not applicable	Art 51
Monthly probability of hospitalization	1.3 (0.04)	1.2-1.4	Normal	Art 51
Monthly probability of ED visits	1.6 (0.03)	1.5-1.7	Normal	Art 51
Proportion of hospitalizations following ED visits (%)	69	55-83	Beta	Art 51
Length of stay (days)	7.3 (5.4)	5.8-8.8	Normal	Art 51
Age (years)	87.9 (3.6)	80.8-95.0	Normal	Art 51
Costs (€)				
VIGIE-AGE program	2210	Not included	Not applicable	Assumption
Hospitalization–EPOCA (per hospitalization)	3714 (3183)	832-9557	LogNorm^f^	Art 51
Hospitalization–SOC (per hospitalization)	3773 (2721)	1056-8873	LogNorm	Art 51
Hospitalization following ED visit	4249 (6373)	395-14,057	LogNorm	Art 51
ED visits	52 (5)	44-61	LogNorm	[16,17]
Outpatient care–EPOCA (per year)	5820 (8111)	4656-6984	Gamma	Art 51
Outpatient care–SOC (per year)	5337 (7759)	4270-6404	Gamma	Art 51
Utility				
General population aged ≥75 years	0.8	Not included	Not applicable	[18]
Hospitalization (per day)	–0.0013 (0.0003)	–0.0016 to –0.0010	Beta	[19]

a PSA: probabilistic sensitivity analysis.

b DSA: deterministic sensitivity analysis.

c SOC: standard of care.

d ED: emergency department.

e Art 51: Vigie-Age Article 51 pilot study.

f LogNorm: Lognormal distribution.

### Key Assumptions

The EPOCA intervention was associated with a reduction in the frequency of ED visits and hospitalizations, based on the results of the Vigie-Age Article 51 study, which showed a significant reduction in the annual number of hospitalizations (4.4, SD 5.1 vs 2.4, SD 4.2) and emergency visits (3.4, SD 4.2 vs 1.6, SD 2.7) before and during the EPOCA intervention.

The probability of hospitalization after an ED visit and the average length of hospital stay were considered similar for both the EPOCA intervention and control groups based on a similar rate of hospitalization after ED visits (3.7, SD 3.3 vs 3.5, SD 2.8) and a similar average length of stay per hospitalization (7.3 days vs 7.4 days) before and during the EPOCA intervention.

Participants in the EPOCA arm were assumed to retain the intervention’s benefits even after they exit the program based on similar results for hospitalization rates when comparing before and during the EPOCA intervention (4.4, SD 5.1 vs 2.4, SD 4.2; average follow-up of 7.5 months; n=269, 66%) and before and after the end of the EPOCA intervention (3.7, SD 5.1 vs 1.4, SD 2.8; average follow-up of 5.2 months; n=69, 17%). This assumption was also based on the expectation that the supportive care established during the intervention, along with the identification and management of potential risk situations and coordination among caregivers facilitated by EPOCA, would generally be sustained following the conclusion of the intervention. Sensitivity analyses were performed by waning the benefit of the EPOCA intervention after 0, 3, and 6 months after the end of the intervention.

It is specifically assumed that patients enrolled in the EPOCA intervention during an ED visit will avoid immediate hospitalization based on the results of the EPOCA intervention, showing that, for patients included in the ED, no immediate hospitalization was observed.

### Costs and Utilities

The analysis included only direct medical costs, encompassing hospitalizations, ED visits, outpatient care, and the EPOCA intervention. Hospitalization and outpatient costs were derived from average expenditures observed in the Vigie-Age Article 51 pilot study and were stratified by study arm to reflect differences in the type of hospitalizations and intensity of outpatient follow-up.

Hospitalization costs were based on production costs (Echelle Nationale des Coûts), which were directly used for the collective perspective. For the payer (NHI) perspective, tariffs were estimated to be 1.27 times lower than production costs, based on observed differences between actual costs and reimbursements [20,21]. Similarly, outpatient care costs were based on tariffs. For the NHI perspective, it was assumed, according to reimbursement rules, that 65% of outpatient costs were reimbursed by the NHI, while the full amount was considered under the collective perspective.

Costs of ED visits were estimated using the average cost per visit, calculated from the total annual funding allocated to emergency services in France [16] and the national number of ED visits [17]. From the NHI perspective, costs were applied only to ED visits that did not result in hospitalization, as the latter were considered part of inpatient costs.

The cost of the EPOCA intervention was based on the reimbursement tariff used in the Vigie-Age Article 51 pilot study, with a mean cost of €2,210 per participant per year. A higher value of €4,441 was used in sensitivity analyses. This cost was applied at the beginning of each year to the proportion of patients remaining in the program. Program duration was assumed to be 6 months for 59% of participants and 12 months for the remaining 41%, resulting in an average duration of 8.4 months. This was implemented as a probability of continued participation, with an estimated 22% of participants still enrolled at the beginning of year 2.

Health utilities were sourced from published literature, using age-adjusted baseline utilities for the general population [18] and applying disutility weights for each day spent hospitalized [19].

### Outcomes and Analysis

The analysis compared discounted (at 2.5% per year [15]) and undiscounted clinical outcomes, including the mean number of hospitalizations per patient, total hospital stay duration, and the mean number of ED visits leading to hospital admissions, between the EPOCA intervention and SOC. Economic outcomes (€2025; EUR €1=US $1.13) included mean hospitalization costs per patient, ED visit costs, outpatient care costs, and intervention costs. Quality of life outcomes were measured in quality-adjusted life years (QALYs).

A cost-utility analysis was conducted, with the primary outcome expressed as the incremental cost-effectiveness ratio, defined as the additional cost per QALY gained with EPOCA compared to SOC.

To evaluate the robustness of the model results, both deterministic and probabilistic sensitivity analyses (PSAs) were performed. Deterministic sensitivity analysis explored the impact of key parameters, including costs, transition probabilities, and utility values, by varying them within their 95% CIs or by +20% to −20% when CIs were unavailable. Results were presented using a tornado diagram to identify the most influential parameters.

PSA was conducted using Monte Carlo simulations to simultaneously assess uncertainty across multiple parameters. Probability distributions were assigned to cost inputs, transition probabilities, and utility values. Results are presented using cost-effectiveness acceptability curves. France does not apply a single official willingness-to-pay threshold; therefore, we used €30,000 per QALY as a commonly used reference value for interpretability of the PSA results.

### Ethical Considerations

This cost-effectiveness and budget impact analysis is a secondary economic modeling study using aggregated, anonymized data from the French Vigie-Age Article 51 study (law 2017-1836) [22]. As no primary data were collected and no direct patient contact occurred, this analysis was exempted from further institutional ethical review. According to French regulations, this type of secondary analysis does not require approval from a research ethics committee, as it falls outside the scope of research involving human participants governed by the French Public Health Code (Law No. 2012-300 of March 5, 2012, known as the “Loi Jardé”) [23].

## Results

The results of the cost-effectiveness and budget impact models, including projections on hospitalizations, ED visits, and direct and indirect costs, are presented in Table 2.

**Table 2.** Cost-effectiveness and budget impact results over a 10-year time horizon with a 2.5% discount rate.

Category	EPOCA, mean (SD)	Standard of care, mean (SD)	Difference (% reduction)
Resource use			
Emergency department visits (n)	2.8 (1.3)	6.1 (2.7)	−3.3 (54)
Hospitalization (n)	4.2 (1.9)	7.8 (3.5)	−3.6 (46)
Hospitalization days	30.6 (27.8)	55.6 (51.7)	−25.0 (45)
Undiscounted costs per patient (€)			
Payer (NHI)^a^ perspective			
Program	2850 (214)	0 (0)	Not applicable
Emergency department visits	145 (69)	315 (148)	−170 (54)
Hospitalization	12,262 (12,758)	23,226 (24,204)	−10,964 (47)
Outpatient care	10,277 (18,030)	9424 (17,210)	853 (−9)
Total	25,535 (23,853)	32,965 (31,927)	−7430 (23)
Collective perspective			
Program	2850 (212)	0 (0)	Not applicable
Emergency department visits	465 (217)	1008 (469)	−543 (54)
Hospitalization	15,573 (15,055)	29,497 (28,562)	−13,924 (47)
Outpatient care	10,277 (17,383)	9424 (16,550)	853 (−9)
Total	29,165 (24,536)	39,929 (34,856)	−10,764 (27)
Cost-effectiveness			
Payer (NHI) perspective			
Discounted QALY^b^	1.30 (0.58)	1.26 (0.57)	0.04
Discounted total costs (€)	24,683 (22,465)	31,792 (30,033)	−7108
ICER^c^	Dominant	Dominated	Not applicable
Collective perspective			
Discounted QALY	1.30 (0.58)	1.26 (0.57)	0.04
Discounted total costs (€)	28,181 (23,171)	38,510 (33,021)	−10,329
ICER	Dominant	Dominated	Not applicable

a NHI: National Health Insurance.

b QALY: quality-adjusted life year.

c ICER: incremental cost-effectiveness ratio.

Over a lifetime horizon, accounting for background mortality, the model estimated an undiscounted average survival of 1.8 years per patient, with an average duration in the EPOCA program of 8.1 months. During this period, patients in the EPOCA arm experienced an average of 2.8 ED visits and 4.2 hospitalizations, compared to 6.1 ED visits and 7.8 hospitalizations in the SOC arm, representing reductions of 54% and 46%, respectively. The decrease in hospitalizations translated into a reduction in the total number of hospital days, from 55.6 days in the SOC arm to 30.6 days in the EPOCA arm.

From the collective perspective, the undiscounted total cost per patient was estimated at €29,165 in the EPOCA arm, compared to €39,929 in the SOC arm. This difference was driven primarily by reductions in hospitalization (€13,924 in savings) and ED visit costs (€543 in savings), partially offset by an increase of €853 in outpatient care costs. After incorporating the program cost (€2,850), the net savings amounted to €10,764 per patient. Similar cost savings were observed under the NHI perspective, with total savings of €7,430 per patient. These included €10,964 in reduced hospitalization costs, €170 in ED savings, and an €853 increase in outpatient costs, with program costs assumed equal across perspectives. Importantly, EPOCA remained cost-saving even when applying the higher intervention cost of €4,441 used in sensitivity analyses.

In terms of health outcomes, the EPOCA arm was associated with 1.30 discounted QALYs compared to 1.26 QALYs in the SOC arm, a gain of 0.04 QALYs, primarily attributable to reduced hospitalizations and associated improvements in quality of life. Discounted total costs were €24,683 for EPOCA and €31,792 for SOC from the NHI perspective, yielding net savings of €7,108. Given that EPOCA resulted in both lower costs and improved outcomes, it was considered a dominant strategy compared to SOC. Similar conclusions were observed in the collective perspective, with €10,329 in total cost savings. EPOCA remained dominant at the higher intervention cost of €4,441 in both perspectives.

**Figure 1.**
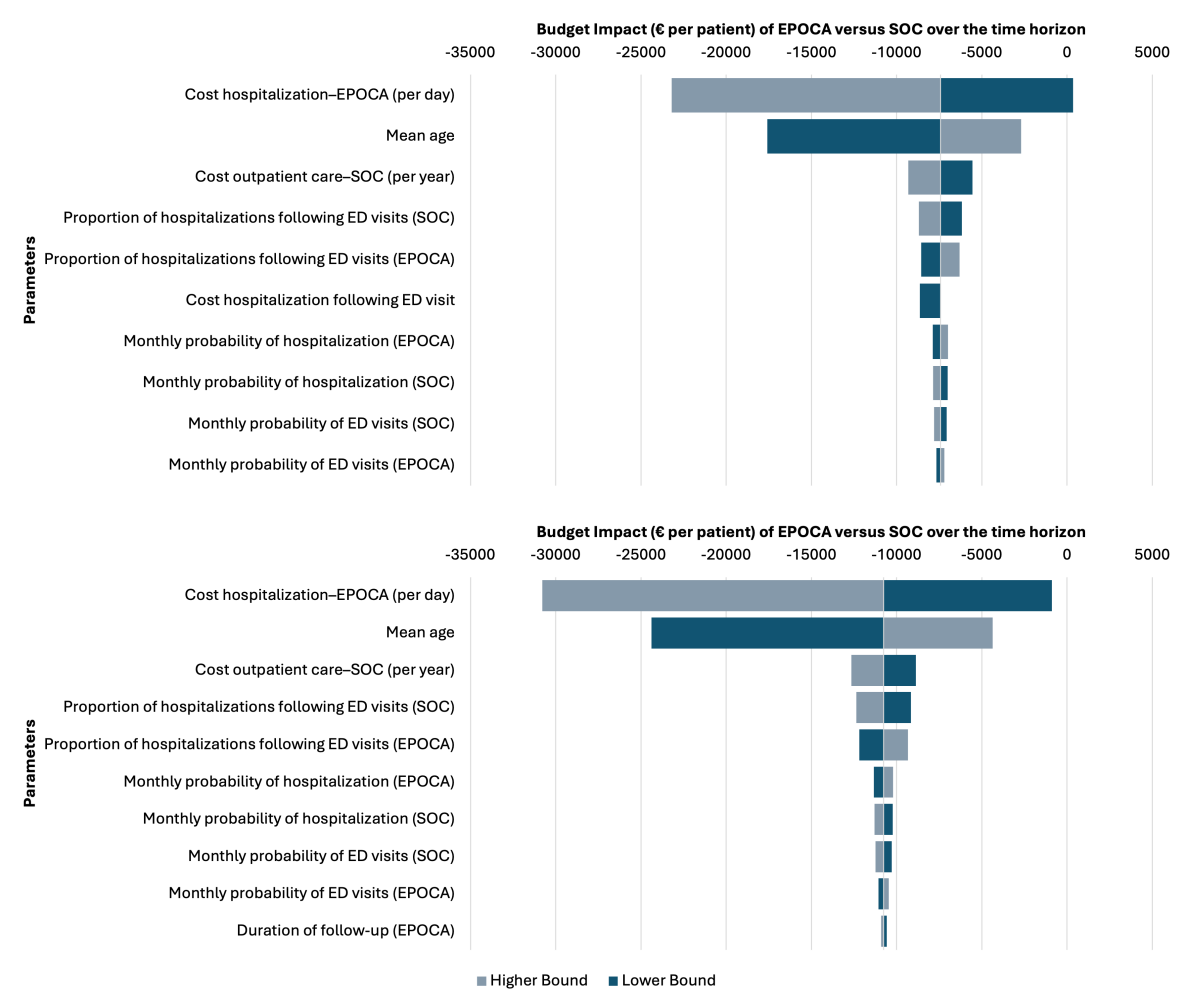
Deterministic sensitivity analysis results. Collective perspective (above) and payer (National Health Insurance) perspective (below). ED: emergency department; SOC: standard of care.

Sensitivity analyses considering no benefit or waning benefit of EPOCA after the end of the intervention did not change the conclusion, with EPOCA remaining a dominant strategy (Table 3). Deterministic sensitivity analysis (Figure 1) identified the average cost of hospitalization as the most influential parameter in the NHI perspective and the mean age at inclusion and the cost of hospitalization as the most influential parameters in the collective perspective. However, even under lower hospitalization cost scenarios, EPOCA remained cost-saving or cost-neutral, confirming the robustness of the model’s conclusions across parameter ranges.

**Figure 2.**
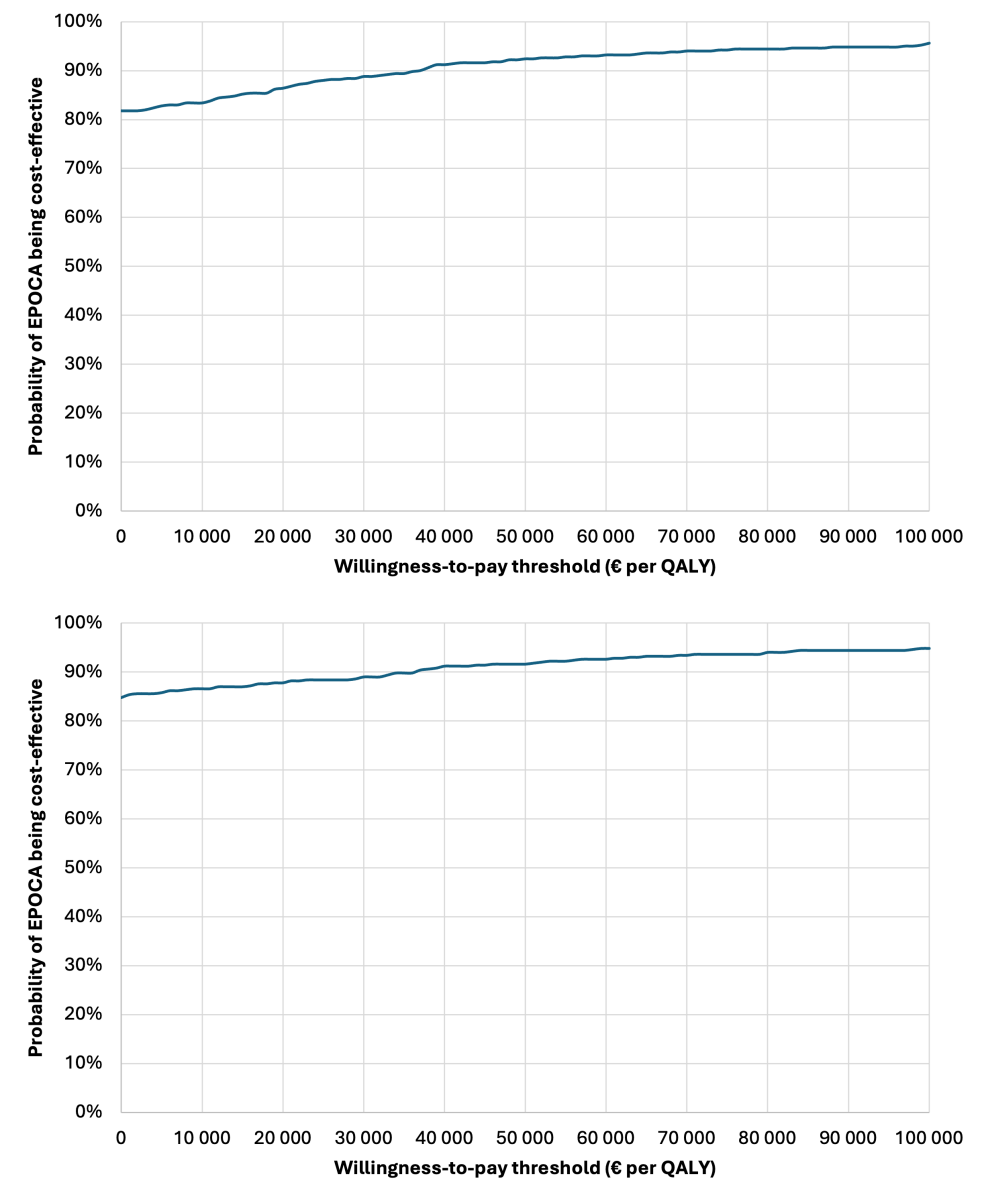
Probabilistic sensitivity analysis results. Collective perspective (above) and payer (National Health Insurance) perspective (below). QALY: quality-adjusted life year.

PSA (Figure 2) further supported these findings. From the collective perspective, EPOCA had a 90% probability of being the dominant strategy and a 95% probability of being cost-effective at a willingness-to-pay threshold of €30,000 per QALY. From the NHI perspective, these probabilities were 80% and 87%, respectively.

**Table 3.** Sensitivity analysis for cost-effectiveness and budget impact results including waning of the program effect, in the payer (National Health Insurance) perspective over a 10-year time horizon with a 2.5% discount rate.

Scenarios	EPOCA, mean (SD)	Standard of care, mean (SD)	Difference (% reduction)
100% waning at the end of the intervention			
Resource use			
Emergency department visits (n)	3.9 (1.3)	6.1 (2.6)	−2.2 (36)
Hospitalization (n)	5.3 (1.9)	7.8 (3.2)	−2.5 (33)
Hospitalization days	38.5 (28.9)	55.6 (43.2)	−17.3 (31)
Cost-effectiveness			
Discounted QALY^a^	1.28 (0.56)	1.26 (0.55)	0.02
Discounted total costs (€)	27,880 (21,539)	31,792 (28,240)	−3912
ICER^b^	Dominant	Dominated	Not applicable
100% waning after 3 months after the end of the intervention			
Resource use			
Emergency department visits (n)	3.5 (1.3)	6.1 (2.7)	−2.6 (42)
Hospitalization (n)	4.9 (1.9)	7.8 (3.4)	−2.9 (37)
Hospitalization days	35.9 (38.7)	55.6 (62.8)	−19.7 (35)
Cost-effectiveness			
Discounted QALY	1.29 (0.58)	1.26 (0.56)	0.03
Discounted total costs (€)	26,833 (22,354)	31,792 (29,975)	−4959
ICER	Dominant	Dominated	Not applicable
100% waning after 6 months after the end of the intervention			
Resource use			
Emergency department visits (n)	3.1 (1.2)	6.1 (2.6)	−3.0 (49)
Hospitalization (n)	4.5 (1.8)	7.8 (3.3)	−3.3 (42)
Hospitalization days	33.0 (31.5)	55.6 (54.9)	−22.6 (41)
Cost-effectiveness			
Discounted QALY	1.29 (0.56)	1.26 (0.55)	0.03
Discounted total costs (€)	25,637 (24,171)	31,792 (34,030)	−6155
ICER	Dominant	Dominated	Not applicable

a QALY: quality-adjusted life year.

b ICER: incremental cost-effectiveness ratio.

## Discussion

This cost-effectiveness analysis shows that the EPOCA RMS could be associated with a reduction in avoidable hospitalizations and ED visits in frail, polypathological older adults. Over a modeled lifetime horizon, EPOCA was associated with a 54% reduction in ED visits and a 46% reduction in hospitalizations, resulting in a 25-day decrease in cumulative hospital stay per patient. These reductions were associated with cost savings of €10,764 per patient from the collective perspective and €7430 from the French payer (NHI) perspective. Notably, even under conservative assumptions for the program costs and in sensitivity analyses, EPOCA remained a dominant strategy, offering better outcomes at lower costs.

These results are consistent with the Article 51 pilot study and real-world data from retrospective observational studies of the EPOCA system [24]. In both hospital-led and GP-led implementations, EPOCA was associated with sharp declines in hospitalizations (−48% to −57%), ED visits (−48% to −62%), and hospital stay duration (−49% to −63%) within just 1 year of follow-up.

International evidence supports the observed outcomes of EPOCA. Similar RMSs have demonstrated reductions in hospital use, with relative risk reductions of up to 57%, 28% in unplanned hospitalizations, and 33% in ED visits [7,9-11,13,25]. EPOCA appears to achieve higher magnitudes of effect, possibly due to its integration into a comprehensive care coordination model involving both hospital and community actors, including nurses, GPs, and geriatricians. Additionally, unlike some RMSs, which may inadvertently increase ED use due to overalerting [10], EPOCA’s design emphasizes triage, anticipatory care, and direct intervention, which could contribute to a true reduction in ED burden. In terms of cost-effectiveness, international evidence also supports the outcomes observed in this study. Previous evaluations of RMSs have reported per-patient savings ranging from approximately €1000 to €2800 [11,26,27], including program costs, which are on the lower end of the €7400 to €10,800 in net savings estimated in this analysis.

These findings have several important implications for health policy. First, EPOCA’s suggested effectiveness in both clinical and economic dimensions supports its integration into national care pathways for older adults. Second, the program’s potential net savings from the NHI perspective support the case for full reimbursement, particularly in light of demographic aging and increasing chronic disease prevalence. Moreover, EPOCA responds to systemic challenges faced by the French health care system: high hospitalization rates among older adults, underresourced primary care, and fragmented coordination between community-based and hospital-based care. By reducing hospital reliance and enabling safe, structured home care, EPOCA could contribute directly to system efficiency and sustainability.

Despite these promising findings, several limitations must be acknowledged. First, the base data from the Vigie-Age Article 51 pilot study involved limited follow-up, requiring extrapolation through Markov modeling. Although sensitivity analyses and waning assumptions confirmed the robustness of the results, longer-term and clinical trial data will be necessary to validate projections. Additionally, because the effectiveness inputs were based on a pre-post comparison without a control group, biases such as regression to the mean and time-varying confounding may have influenced the results. Although the observed differences are likely too large to be entirely due to bias, smaller differences could affect conclusions. Second, there was significant variability in available hospitalization costs, potentially impacting cost estimates. Third, outpatient cost data were partially incomplete, particularly for nonreimbursed services, which may have led to conservative cost assumptions. Fourth, the disutility values relied on data from heart failure patients [19], which may not be directly applicable to the population used in the model since no more closely matching source was found. Nevertheless, 73% of those in the Vigie-Age Article 51 study presented cardiovascular risk factors, and heart failure hospitalization occurred frequently. Finally, although the program likely reduces caregiver burden through improved coordination and reduced crisis care, these effects were not directly measured and warrant further investigation.

In conclusion, based on currently limited real-world evidence from the Vigie-Age study, this modeling study suggests that the EPOCA RMS may be a cost-effective intervention for managing older patients at high risk of hospitalization. It could not only reduce acute care use but also improve quality-adjusted life expectancy and lower total health care costs. From the NHI perspective, the budget impact may be neutral to favorable. Supported by real-world implementation data and consistent with international best practices, EPOCA has strong potential for national scale-up. Embedding such models within France’s older adult care infrastructure could enhance care quality, reduce hospital pressure, and better address the needs of a rapidly aging population. These results will need to be confirmed with clinical trial results.

## Supplementary material

10.2196/80302 Checklist 1 CHEERS 2022 checklist.

## Abbreviations

ED: emergency department
GP: general practitioner
NHI: National Health Insurance
PSA: probabilistic sensitivity analysis
QALY: quality-adjusted life year
RMS: remote monitoring system
SOC: standard of care
